# Obinutuzumab plus bendamustine in previously untreated patients with CLL: a subgroup analysis of the GREEN study

**DOI:** 10.1038/s41375-018-0146-5

**Published:** 2018-04-27

**Authors:** Stephan Stilgenbauer, Veronique Leblond, Robin Foà, Sebastian Böttcher, Osman Ilhan, Wolfgang Knauf, Eva Mikuskova, Christoph Renner, Eugen Tausch, Dariusz Woszczyk, Ekaterina Gresko, Linda Lundberg, Tom Moore, Thea Morris, Susan Robson, Francesc Bosch

**Affiliations:** 1 Department of Internal Medicine III, Ulm University, Ulm, Germany; 2 Present Address: Klinik für Innere Medizin I, Universitätsklinikum des Saarlandes, Homburg, Germany; 3 UPMC GRC11-GRECHY, AP-HP Hôpital Pitié Salpêtrière, Paris, France; 4 Department of Cellular Biotechnologies and Hematology, ‘Sapienza’ University, Rome, Italy; 5 Second Department of Medicine, University of Schleswig-Holstein, Campus Kiel, Kiel, Germany; 6 Clinic III, Hematology, Oncology and Palliative Medicine, University of Rostock, Rostock, Germany; 7 Ankara University, Ankara, Turkey; 8 Onkologische Gemeinschaftspraxis, Agaplesion Bethanien Krankenhaus, Frankfurt, Germany; 9 National Cancer Institute, Bratislava, Slovakia; 10 OnkoZentrum Zürich, Zürich, Switzerland; 11 State Hospital, Opole, Poland; 12 Present Address: Haematology Department, University of Opole, Provincial Hospital, Opole, Poland; 13 F. Hoffmann-La Roche Ltd, Basel, Switzerland; 14 University Hospital Vall d’Hebron, Barcelona, Spain

## Abstract

GREEN (NCT01905943) is a non-randomized, open-label phase IIIb study investigating obinutuzumab alone or plus chemotherapy in chronic lymphocytic leukemia (CLL). We report a preplanned subgroup analysis of 158 previously untreated CLL patients receiving obinutuzumab–bendamustine (G-B). Patients received six 28-day cycles (C) of G-B: obinutuzumab day (D)1/D2 of C1 (25 mg D1/975 mg D2), 1000 mg D8 and D15 of C1, and D1 of C2–6; and bendamustine 70/90 mg/m^2^ D1 and D2 of C1–6. The primary endpoint was safety/tolerability. Grade ≥3 adverse events (AEs) occurred in 82.3% of patients, including neutropenia (49.4%), thrombocytopenia (12.0%) and febrile neutropenia (10.8%). Serious AEs included neutropenia (12.7%), febrile neutropenia (9.5%) and pneumonia (7.6%). Rates of grade ≥3 infections and infusion-related reactions were 20.3% and 17.1%, respectively. Due to tumor lysis syndrome (TLS; 8.2%), including two associated fatalities (one in another study cohort), additional risk-minimization measures were implemented. Overall response rate was 81.0%. After 32.8 months’ median observation time, 2-year progression-free survival was 81.8%. Minimal residual disease was undetectable in 59.5% (94/158) and 27.8% (44/158) of patients for blood and bone marrow, respectively. Frontline G-B appears to have manageable toxicity with clinical activity in CLL. Careful TLS risk assessment, pretreatment and monitoring is required.

## Introduction

Chronic lymphocytic leukemia (CLL) is the most common adult leukemia in the western world [1]. The standard first-line treatment for physically fit CLL patients is chemoimmunotherapy with fludarabine–cyclophosphamide–rituximab (R-FC) [2–6]. However, many CLL patients are elderly with comorbidities and ineligible for fludarabine-based treatment [7, 8]. New combinations with less toxicity and alternative chemotherapy backbones are needed.

Chemoimmunotherapy with rituximab–bendamustine (R-B) is effective and well tolerated in fit patients with previously untreated or relapsed/refractory CLL [9, 10], although its efficacy was inferior to R-FC in the phase III CLL10 study [5]. The pivotal CLL11 study demonstrated that the glycoengineered, type II anti-CD20 antibody obinutuzumab (GA101) was superior to rituximab (combined with chlorambucil; G-Clb vs R-Clb) in treatment-naive patients with CLL and coexisting conditions [11]. Thus, there is a rationale for evaluating obinutuzumab–bendamustine (G-B) in CLL.

GREEN (NCT01905943) is an ongoing phase IIIb study investigating obinutuzumab alone or with chemotherapy in previously untreated or relapsed/refractory CLL [12]. Here, we report on a preplanned safety and efficacy analysis from a subgroup of previously untreated CLL patients who received G-B in GREEN.

## Patients and methods

### Study design

GREEN is a non-randomized, open-label, non-comparative, multicenter study in previously untreated or relapsed/refractory CLL. The primary objective is to evaluate the safety/tolerability of obinutuzumab alone or combined with various chemotherapy regimens; the secondary objective is to assess efficacy. The exploratory objective is to investigate alternative obinutuzumab administration measures to mitigate/reduce infusion-related reactions (IRRs). For this, patients included in GREEN were assigned to three different cohorts (Supplementary Figure S1).

Planned analyses for GREEN were to report safety and efficacy summaries for each treatment regimen separately as well as pooled overall safety. Here, we report the subgroup of previously untreated patients receiving G-B assigned to cohort 1 of GREEN (trial fully accrued since 30 March 2016 (first enrolled, 24 October 2013), with follow-up ongoing), who received a modified obinutuzumab dosing regimen to mitigate IRRs: the initial dose was given in two parts on consecutive days (25 mg on day (D) 1 of cycle (C) 1 at an infusion rate of 12.5 mg/h, followed by 975 mg on D2 at an initial rate of 50 mg/h, then 1000 mg on D8 and D15 of C1, and D1 of C2–6 as standard).

Chemotherapy options (28-day cycles) in all cohorts were partly dependent on patient fitness and based on investigator choice (Supplementary Figure S1). Bendamustine was dosed at 90 mg/m^2^ (or 70 mg/m^2^ in unfit patients at the investigator’s discretion) on D1 and D2 of C1–6. Granulocyte colony-stimulating factor (G-CSF) use was permitted; primary prophylaxis was advised for patients aged ≥60 years and/or with comorbidities.

Risk-minimization measures (including adequate hydration (fluid intake 3 l/day, starting 1–2 days before first obinutuzumab dose) and allopurinol (or similar e.g. rasburicase) prophylaxis for at least 72 h prior to first obinutuzumab dose) for tumor lysis syndrome (TLS) [13], an identified risk in patients treated with obinutuzumab, were included in the protocol from the start. Patients were considered at risk of TLS if they had node(s) ≥10 cm; or node(s) ≥5 cm and <10 cm, and an absolute lymphocyte count (ALC) ≥25 × 10^9^/l or renal impairment (creatinine clearance (CrCl) <70 ml/min); or ALC ≥25 × 10^9^/l and CrCl <70 ml/min. After two reports of fatal TLS cases in G-B-treated patients (including one in cohort 1), additional risk-minimization measures, including investigator training to emphasize the importance of TLS prophylaxis, were instigated for all cohorts, as described in the supplement. However, these additional measures did not apply to patients in cohort 1 as they had already completed treatment.

GREEN was conducted in accordance with the Declaration of Helsinki, Good Clinical Practice Guidelines and local laws and was approved by institutional review boards/ethics committees at participating centers. Patients provided written informed consent. All authors had access to the primary clinical data and were involved in the data analysis.

### Patient population

Cohort 1 enrolled previously untreated CLL patients only. Inclusion criteria were: age ≥18 years; requiring treatment per International Workshop on CLL (iwCLL) criteria; [14] adequate hematologic function; and Eastern Cooperative Oncology Group performance status ≤2. Patients with a 17p deletion could be enrolled; those with severe renal impairment (CrCl <30 ml/min) were excluded. Full inclusion/exclusion criteria for previously untreated CLL patients (cohorts 1–3) are detailed in the supplement.

### Study endpoints

The primary endpoint of GREEN was safety/tolerability. Secondary endpoints were overall response rate (ORR) including complete response (CR), progression-free survival (PFS) and minimal residual disease (MRD).

### Assessments

#### Safety

Adverse events (AEs), serious AEs (SAEs) and AEs of special or particular interest (AESI/AEPI) were monitored and graded using National Cancer Institute Common Terminology Criteria for AEs version 4.0. IRRs were defined as any AE occurring during or within 24 h of obinutuzumab infusion and considered related to obinutuzumab.

#### Efficacy

ORR was assessed by the investigator per iwCLL criteria [14] at the ‘final response assessment’ visit (scheduled 84 days after last treatment dose). A computed tomography (CT) scan was required to confirm CR and partial response (PR); patients lacking a valid CT scan were classed as stable disease (SD). A bone marrow biopsy was required for confirmation of CR; patients otherwise meeting CR criteria but lacking a valid biopsy were classed as PR. Other efficacy assessments including PFS, MRD measurement and analysis of prognostic markers are detailed in the supplement.

#### Statistical methodology

Details of the sample size estimation are provided in the supplement. Safety variables were analyzed in the safety population (*N* = 158), i.e., all patients who received at least one dose of study treatment. Efficacy variables were analyzed in the intent-to-treat (ITT) population (*N* = 158), comprising all patients from cohort 1 who received at least a partial dose of both obinutuzumab and bendamustine (data cut-off, 29 December 2016). For the MRD analyses, the ‘intent-to-ship’ population (*N* = 140) comprised all patients whose MRD samples at the final response assessment could be shipped to the central laboratory within 48 h (thus excluding Argentina, Brazil, Canada, China, Korea, Mexico, Thailand and Uruguay). The ‘MRD-evaluable’ population (*N* = 105) comprised all patients with an available MRD result (blood or bone marrow).

For ORR, patients with a missing response assessment during the defined time window (D56–168 after last treatment dose) were considered non-responders. Response rates were presented with two-sided, 95% Clopper–Pearson confidence intervals (CIs). Estimates for the survival functions for PFS were obtained using the Kaplan–Meier approach. After the data snapshot was taken for analysis, a further 5 AEs related to 4 patients in this subgroup were reported late by the sites on the database that remained open to continue collecting information until the final analysis. In addition, one patient with SD at the final response assessment had this changed to PR. These updates are not part of the statistical analysis summary tables and listings presented. Please see Supplementary Tables S1 and S2.

## Results

### Patient, treatment exposure and observation time

The ITT population comprised 158 patients with CLL (70 fit, 88 unfit) (Table 1; Supplementary Figure S2). Mean number of obinutuzumab administrations was 8.4 (planned, 9) and 149 (94.3%) patients received ≥90% of the planned dose (based on actual/planned doses). Mean number of bendamustine cycles was 5.3 (planned, 6), with 142 (89.9%) patients receiving ≥90% of the planned dose (68 patients received bendamustine 70 mg/m^2^ and 90 patients received 90 mg/m^2^). Median observation time was 32.8 (range, 0.5–37.5) months.

**Table 1:** Baseline patient characteristics in patients receiving G-B in cohort 1 of the GREEN study (intent-to-treat population)

Characteristic	All patients (*N* = 158)
Median age, years (range)	69.0 (42–83)
Male/female, *n* (%)	103/55 (65.2/34.8)
CIRS >6, *n* (%)	28 (17.7)
CrCl <70 ml/min, *n* (%)	73 (46.2)
CrCl <50 ml/min, *n* (%)	21 (13.3)
CIRS >6 and CrCl <70 ml/min, *n* (%)	13 (8.2)
Fitness, *n* (%)	
Fit^a^	70 (44.3)
Unfit^b^	88 (55.7)
Binet stage at screening, *n* (%)	
A	48 (30.4)
B	57 (36.1)
C	53 (33.5)
Absolute lymphocyte count, *N* = 155, *n* (%)	
≥50 × 10^9^/l	88 (56.8)
Tumor bulk, *N* = 139, *n* (%)	
≥5 cm	95 (68.3)
Genomic aberrations, *N* = 146, *n* (%)^c^	
17p deletion	11 (7.5)
11q deletion	26 (17.8)
12q trisomy	26 (17.8)
13q deletion	52 (35.6)
Other abnormality	6 (4.1)
Normal	25 (17.1)
IGHV, *N* *=* 136, *n* (%)	
Unmutated	92 (67.6)
Mutated	44 (32.4)
ZAP70, *N* = 129, *n* (%)	
Positive	82 (63.6)
Negative	47 (36.4)
CD38, *N* = 129, *n* (%)	
Positive	70 (54.3)
Negative	59 (45.7)

*CIRS* Cumulative Illness Rating Scale, *CrCl* creatinine clearance, *G-B* obinutuzumab plus bendamustine, *IGHV* immunoglobulin heavy variable chain

^a^ CIRS ≤6 and CrCl ≥70 ml/min

^b^ CIRS >6 and/or CrCl <70 ml/min

^c^ According to the hierarchical model of genomic aberrations [29]

### Safety

Most patients (96.8%, 153/158) reported at least one AE; neutropenia (62.7%), pyrexia (41.8%) and thrombocytopenia (32.3%) were the most frequent. Overall, 88.6% of patients experienced at least one treatment-related AE, most commonly neutropenia (57.0%), pyrexia (31.0%) and thrombocytopenia (29.1%). Twenty-six patients (16.5%) discontinued at least one study treatment prematurely due to an AE, 11 (7.0%) of whom did so because of neutropenia. G-CSF was administered to 107/158 (67.7%) patients at least once during the study. Common grade ≥3 AEs, serious AEs and grade ≥3 AESI are summarized in Table 2.

**Table 2:** Grade 3 or higher adverse events and serious adverse events in patients receiving G-B in cohort 1 of the GREEN study (safety population)

*n* (%)	All patients (*N* = 158)	Fit patients^a^ (*n* = 70)	Unfit patients^b^ (*n* = 88)
Grade ≥3 AEs (reported by ≥2% patients in overall population) by preferred term			
Any	130 (82.3)	52 (74.3)	78 (88.6)
Neutropenia	78 (49.4)	34 (48.6)	44 (50.0)
Thrombocytopenia	19 (12.0)	8 (11.4)	11 (12.5)
Febrile neutropenia	17 (10.8)	8 (11.4)	9 (10.2)
Lymphopenia	14 (8.9)	6 (8.6)	8 (9.1)
Anemia	13 (8.2)	3 (4.3)	10 (11.4)
Leukopenia	13 (8.2)	6 (8.6)	7 (8.0)
Tumor lysis syndrome	13 (8.2)	2 (2.9)	11 (12.5)
Pneumonia	12 (7.6)	5 (7.1)	7 (8.0)
Hypertension	11 (7.0)	5 (7.1)	6 (6.8)
Hyperglycemia	6 (3.8)	4 (5.7)	2 (2.3)
Squamous cell carcinoma	5 (3.2)	1 (1.4)	4 (4.5)
Diarrhea	4 (2.5)	0	4 (4.5)
Hyperuricemia	4 (2.5)	4 (5.7)	0
Serious AEs (reported by ≥2% patients in overall population) by preferred term			
Any	96 (60.8)	37 (52.9)	59 (67.0)
Neutropenia	20 (12.7)	9 (12.9)	11 (12.5)
Febrile neutropenia	15 (9.5)	6 (8.6)	9 (10.2)
Pneumonia	12 (7.6)	5 (7.1)	7 (8.0)
Pyrexia	11 (7.0)	4 (5.7)	7 (8.0)
Tumor lysis syndrome	6 (3.8)	0	6 (6.8)
Squamous cell carcinoma	4 (2.5)	1 (1.4)	3 (3.4)
Thrombocytopenia	4 (2.5)	1 (1.4)	3 (3.4)
Grade ≥3 AESI/AEPI^c^			
Neutropenia	84 (53.2)	37 (52.9)	47 (53.4)
Infections	32 (20.3)	11 (15.7)	21 (23.9)
IRRs	27 (17.1)	9 (12.9)	18 (20.5)
Thrombocytopenia	19 (12.0)	8 (11.4)	11 (12.5)
Second malignancies	13 (8.2)	4 (5.7)	9 (10.2)
Tumor lysis syndrome	13 (8.2)	2 (2.9)	11 (12.5)

*AE* adverse event, *AEPI* adverse events of particular interest, *AESI* adverse events of special interest, *CIRS* Cumulative Illness Rating Scale, *CrCl* creatinine clearance, *G-B* obinutuzumab plus bendamustine, *IRR* infusion-related reaction, *MedDRA* Medical Dictionary for Regulatory Activities

^a^ CIRS ≤6 and CrCl ≥70 ml/min

^b^ CIRS >6 and/or CrCl <70 ml/min

^c^ IRRs were defined as any AE occurring during or within 24 h of obinutuzumab infusion and considered related to obinutuzumab; infection selection was via the MedDRA system order class ‘Infections and Infestations’; second malignancy selection was via the MedDRA system organ class ‘Neoplasms Benign, Malignant, and Unspecified’ starting 6 months after the first study drug intake; neutropenia and thrombocytopenia selection was via their MedDRA basket dataset subgroups; and tumor lysis syndrome was defined by its preferred term

IRRs occurred in 57.6% (*n* = 91) of patients; 17.1% (*n* = 27) experienced grade ≥3 IRRs. Serious IRRs occurred in 10.1% (*n* = 16) of patients (no fatal cases). Infections occurred in 54.4% (*n* = 86) of patients; 20.3% reported grade ≥3 infection. Grade ≥3 infections (by preferred term) in more than one patient were: pneumonia (*n* = 12); sepsis and urinary tract infection (*n* = 3 each); and cytomegaloviral pneumonia, erysipelas, herpes zoster and lung infection (*n* = 2 each). Serious infections occurred in 19.6% of patients, including (≥1%): pneumonia (*n* = 12); sepsis (*n* = 3); and herpes zoster, lung infection, cytomegaloviral pneumonia and urinary tract infection (*n* = 2 each). Grade ≥3 AEs related to neutropenia were reported by 53.2% of patients. TLS (grade ≥3) was observed in 13 (8.2%) patients, and included one fatality. TLS was one of the most common SAEs (*n* = 6; 3.8%) by preferred term, along with neutropenia (12.7%), febrile neutropenia (9.5%), pneumonia (7.6%) and pyrexia (7.0%) (Table 2).

The tolerability of G-B appeared more favorable in fit vs unfit patients based on rates of grade ≥3 AEs and SAEs (Table 2). Rates of grade ≥3 TLS, infections and IRRs were numerically lower in fit vs unfit patients. Serious TLS events occurred in 6.8% of unfit patients, but were not observed in fit patients.

Seventeen deaths (10.8%) were reported (fit *n* = 6; unfit *n* = 11), two of which occurred on, or within 28 days of last, study treatment; five deaths (all resulting from AEs) were considered related to treatment (West Nile virus infection, acute liver failure, febrile neutropenia with TLS, arrhythmia and brain metastasis of adenocarcinoma). Four deaths were due to disease progression and 13 were due to AEs (two with prior progressive disease (PD]) (Supplementary Table S3).

### Response assessment

ORR at the final response assessment was 81.0% (95% CI, 74.0–86.8; CR (including CR with incomplete marrow recovery (CRi)), 34.8%; Table 3). Overall, 9.5% of patients had a missing assessment and were classed as non-responders. ORR was broadly similar in unfit and fit patients (Table 3), and of those patients who reported TLS in the ITT population, 69.3% (9/13) responded to treatment. Response rates were also similar between bendamustine dose groups (70 mg/m^2^: ORR, 83.8% (57/68; 95% CI, 72.9–91.6); CR/CRi, 38.2% (26/68); 90 mg/m^2^: ORR, 78.9% (71/90; 95% CI, 69.0–86.8); CR/CRi, 32.2% (29/90)).

**Table 3:** Response in patients receiving G-B in cohort 1 of the GREEN study at final response assessment (intent-to-treat population)

Response, *n* (%)	All patients (*N* = 158)	Fit patients^a^ (*n* = 70)	Unfit patients^b^ (*n* = 88)
Overall response	128 (81.0)	60 (85.7)	68 (77.3)
Complete response^c^	55 (34.8)	23 (32.9)	32 (36.4)
Partial response	73 (46.2)	37 (52.9)	36 (40.9)
Stable disease	13 (8.2)	4 (5.7)	9 (10.2)
Progressive disease	2 (1.3)	1 (1.4)	1 (1.1)
Failure (due to missing assessment)	15 (9.5)	5 (7.1)	10 (11.4)

*CIRS* Cumulative Illness Rating Scale, *CrCl* creatinine clearance, *G-B* obinutuzumab plus bendamustine

^a^ CIRS ≤6 and CrCl ≥70 ml/min

^b^ CIRS >6 and/or CrCl <70 ml/min

^c^ Including complete response with incomplete marrow recovery

**Fig. 1:**
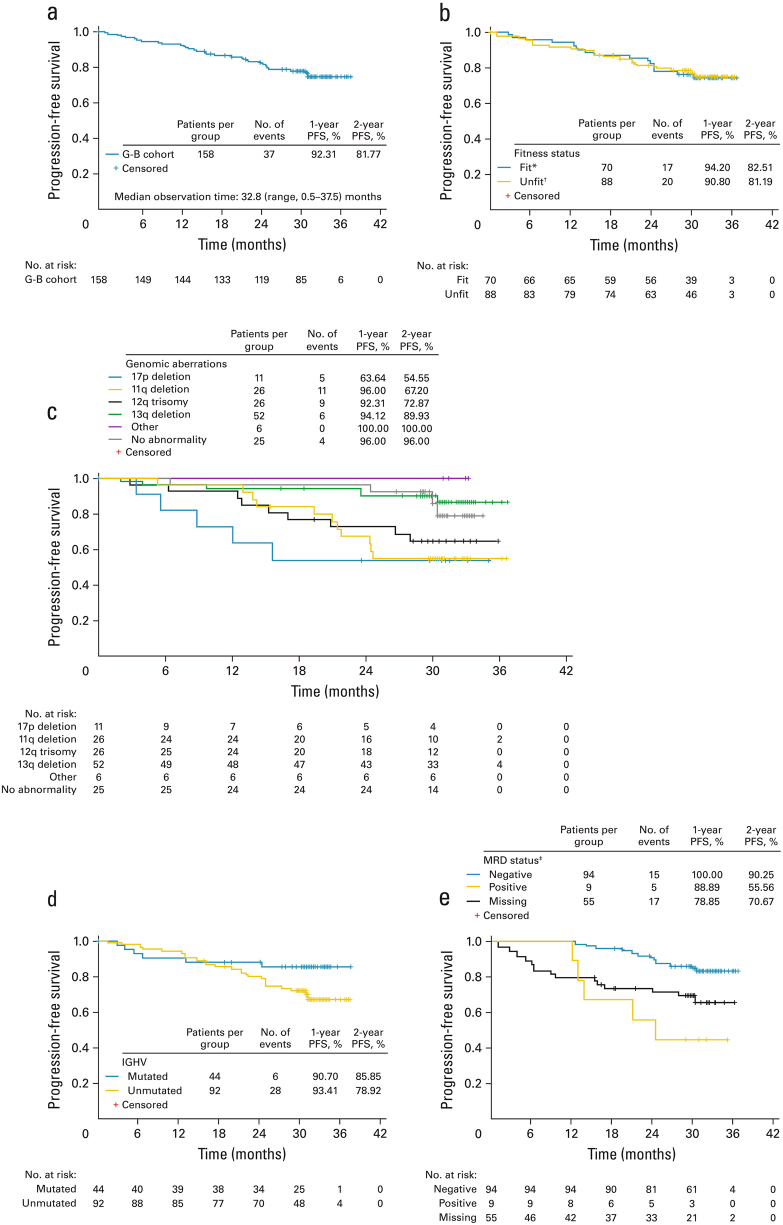
Progression-free survival in patients receiving G-B in cohort 1 of GREEN: **a** in the overall study population; **b** in fit vs unfit patients; **c** by genomic aberrations, according to the hierarchical model [29]; **d** by IGHV mutation status; and **e** by MRD status at final response assessment in blood (intent-to-treat population). *CIRS ≤6 and CrCl≥ 70 ml/min; ^†^CIRS >6 and/or CrCl <70 ml/min; ^‡^these data should be interpreted with caution as the subgroups are based on a study outcome, not baseline characteristics. There was also a low number of events (*n* = 20) and small number of MRD-positive patients (*n* = 9). The MRD-evaluable subgroup comprised patients who did not progress or die and had an MRD result available at the final response assessment in either blood or bone marrow (*n* = 105). The time window for MRD assessment was 77 to 168 days after last treatment. The MRD-‘missing’ subgroup included 54 patients. In addition, 1 out of 105 patients had a result available only in bone marrow and therefore came out as ‘missing’ in the blood population (therefore the ‘missing’ group in **e** is *n* = 55 and not 54). Reasons for ‘missing’ included sample not taken (*n* = 20), shipment could not take place within 48 h (*n* = 18), measurement was outside the time window for the final response assessment (*n* = 9) or other reason (*n* = 7). Eleven patients with missing MRD status (in blood and bone marrow) progressed (*n* = 5) or died (*n* = 6) within the period since last treatment dose up to 168 days. CIRS Cumulative Illness Rating Scale, CrCl creatinine clearance, G-B obinutuzumab plus bendamustine, IGHV immunoglobulin heavy variable chain, MRD minimal residual disease, PFS progression-free survival

Median PFS was not reached; at data cut-off, 23.4% of patients had experienced an event (Fig. 1a). Estimated PFS at 12 and 24 months was 92.3% and 81.8%, respectively. PFS appeared similar in fit vs unfit patients (Fig. 1b). Despite the limited size of subgroups, there was a trend for shorter PFS in older patients (≥65 years), and in patients with a 17p or 11q deletion, 12q trisomy, unmutated immunoglobulin heavy variable chain (IGHV) or CD38^+^ CLL (Supplementary Table S4; Fig. 1c, d).

### MRD analysis at final response assessment

For the ITT population, undetectable MRD (<10^-4^, i.e., MRD-negativity (MRD^–^)) was observed in 59.5% (94/158) and 27.8% (44/158) for blood and bone marrow samples, respectively. MRD^–^ rates in the intent-to-ship population were 67.1% (94/140; blood) and 31.4% (44/140; bone marrow). The MRD-evaluable population comprised 104 patients with an evaluable blood sample and 65 with an evaluable bone marrow sample (64 with blood and bone marrow and 1 with bone marrow only). MRD^–^ rates in the MRD-evaluable population were 90.4% (94/104) and 67.7% (44/65) for blood and bone marrow, respectively (Table 4).

**Table 4:** Disease characteristics and response (MRD or clinical) at the final response assessment in patients receiving G-B in cohort 1 of the GREEN study

Factor, *n*/*N* (%) patients	MRD negative^a^ (blood)	MRD negative^a^ (bone marrow)	CR/Cri^b^
All patients	94/104 (90.4)	44/65 (67.7)	55/158 (34.8)
Binet stage A	32/32 (100)	13/18 (72.2)	19/48 (39.6)
Binet stage B+C	62/72 (86.1)	31/47 (66.0)	36/110 (32.7)
Disease bulk ≥5 cm	57/64 (89.1)	27/39 (69.2)	29/95 (30.5)
ALC ≥50 × 10^9^/l	52/59 (88.1)	25/38 (65.8)	33/88 (37.5)
17p deletion	3/6 (50.0)	2/3 (66.7)	2/11 (18.2)
11q deletion	18/23 (78.3)	5/15 (33.3)	7/26 (26.9)
12q trisomy	18/20 (90.0)	12/14 (85.7)	8/26 (30.8)
13q deletion	32/32 (100)	13/19 (68.4)	22/52 (42.3)
CD38 positive	49/55 (89.1)	17/28 (60.7)	22/70 (31.4)
CD38 negative	37/41 (90.2)	22/30 (73.3)	22/59 (37.3)
ZAP70 positive	54/60 (90.0)	22/34 (64.7)	30/82 (36.6)
ZAP70 negative	32/36 (88.9)	17/24 (70.8)	14/47 (29.8)
*IGHV* mutated	31/31 (100)	12/17 (70.6)	17/44 (38.6)
*IGHV* unmutated	58/68 (85.3)	28/44 (63.6)	30/92 (32.6)

*ALC* absolute lymphocyte count, *CR* complete response, *CRi* complete response with incomplete marrow recovery, *G-B* obinutuzumab plus bendamustine, *IGHV* immunoglobulin heavy variable chain, *MRD* minimal residual disease

^a^ Patients with evaluable (laboratory sample with a valid result at the final response assessment) MRD

^b^ Patients who achieved CR/CRi at the final response assessment in the intent-to-treat population

MRD^–^ rates were similar for bendamustine dose groups: 92.3% (36/39; 95% CI, 79.1–98.4) and 89.2% (58/65; 95% CI, 79.1–95.6) for 70 and 90 mg/m^2^, respectively.

Patients who were MRD negative in blood at final response assessment had longer PFS than those who were not (Fig. 1e); however, this may be biased as all early withdrawals had no MRD assessment at the final response assessment by definition.

## Discussion

In the phase Ib GALTON and phase II GIBB studies, G-B appeared to have acceptable toxicity and was active in previously untreated CLL patients [15, 16]. Our subgroup analysis of a cohort of first-line patients receiving G-B indicates a similar trend in a larger international, multicenter study, and suggests that G-B may have manageable toxicity and promising efficacy in most patients. However, it also highlights the need for effective monitoring and management of TLS in G-B-treated patients. Thirteen (8.2%) patients herein experienced TLS with one associated death, whereas the incidence of TLS with G-Clb in the pivotal CLL11 trial was 4% with no associated deaths [11]. With the development of increasingly efficacious treatments, recognition and management of TLS in CLL is of ever-greater importance, especially when such treatments are given to patients with comorbidities, such as renal impairment. Specific guidance for the prevention and treatment of TLS and IRRs is provided in the labeling for obinutuzumab [17, 18], and a manuscript reporting the outcome of different IRR risk-mitigation strategies employed in GREEN will be published separately.

In addition, as detailed in the supplement, at-risk patients must be recognized prior to treatment initiation, receive appropriate prophylaxis and be monitored closely for occurrence of TLS signs during initial treatment. Any additional guidelines according to standard practice should be followed.

AEs were consistent with the known safety profile of obinutuzumab. Rapid B-cell depletion with obinutuzumab, accompanied by rapid release of cytokines including interleukin-6, interleukin-8 and tumor necrosis factor alpha, may explain the frequency and intensity of IRRs with obinutuzumab vs rituximab [11, 19]. Grade ≥3 IRRs developed in 20.5% of unfit patients (17.1% of G-B patients overall), which was comparable with that (20%) reported for G-Clb in CLL11 [11]. As with G-Clb in CLL11, no IRRs were fatal. Obinutuzumab administration in this subcohort of GREEN differed from that in CLL11 for the initial 1000 mg, with an alternative split dose (25 mg on D1 at 12.5 mg/h, and 975 mg on D2) [11]. Although split dosing did not appear to substantially reduce the incidence of IRRs vs CLL11, differences in study designs, treatment regimens and patient populations limit this comparison. Grade ≥3 TLS and IRRs, and TLS as an SAE, were more frequently reported in unfit compared with fit patients, highlighting the need to monitor unfit patients and provide appropriate prophylaxis.

Grade ≥3 neutropenia was reported in approximately half of patients, which was higher than that previously reported with G-Clb and R-B, but similar to that seen with G-B and G-FC [9, 11, 16]; importantly, grade ≥3 neutropenia with G-B in the present study was not associated with a marked increase in infections.

Seventeen deaths were reported in this cohort; 13 were due to AEs, of which 5 were considered related to G-B and 2 occurred post PD (Supplementary Table S3). No deaths were reported in the phase Ib GALTON study in fit patients with previously untreated CLL treated with G-B; however, this was a relatively small study (20 patients received G-B) undertaken at select sites [16]. Three deaths were reported among 102 patients in the phase II GIBB trial, but none were deemed related to treatment or CLL [15]. In CLL11, the percentage of patients with a fatal AE was lower with G-Clb (4%) than with rituximab-chlorambucil (R-Clb) or chlorambucil alone (6% and 9%, respectively) [11].

Patients receiving G-B achieved an ORR of 81.0%, with CR/CRi and PR in 34.8% and 46.2% of patients, respectively. Notably, response data were missing for 9.5% of patients (classed as non-responders). Despite some differences in response assessment (timing and criteria) between studies, response rates in all patients were broadly comparable with those reported for R-B in CLL10 (fit patients: ORR 96%, CR 31%) [5], G-Clb in CLL11 (unfit patients: ORR 78.4%, CR 20.7%) [11], and G-B in the GALTON (fit patients: ORR 90%, CR/CRi 45%) [16] and GIBB (ORR 89.2%; CR/CRi 49.0%) [15] studies. Efficacy (response and PFS) was also similar to R-FC in CLL8 (fit patients) [3]. Importantly, response rates were similar between fit and unfit patients, and a high CR rate was noted in patients with Binet stage B+C CLL, showing that G-B is efficacious in this population. The high CR/CRi rate in patients with unmutated IGHV (34.8%) was reassuring given the association between unmutated IGHV status and poor prognosis [20].

MRD status has been identified as a strong post-treatment prognostic factor after chemoimmunotherapy, with MRD^–^ independently associated with superior PFS and overall survival [21–24]. The sensitive quantification of MRD using flow cytometry in patients with CLL is well documented [25–28]. MRD^–^ in blood after G-B is expected to be associated with a favorable course during follow-up, as reported with G-Clb in CLL11 or R-FC in CLL8 [4, 11]. Despite the limited sample size and potential for bias, as patients were categorized based on outcome rather than baseline characteristics, the PFS data obtained so far in the present analysis are suggestive of a favorable prognosis in MRD-negative patients. The apparent discord between the high rate of MRD^–^ but slightly lower than expected CR rates likely reflects the rigorous iwCLL assessment criteria [14] used for response classification and that patients with missing assessments within the required time window were down-classed.

Genomic factors, such as 11q and 17p chromosome deletions, mutated *TP53*, unmutated IGHV status, ZAP70 expression, and markers including increased serum β_2_-microglobulin, are associated with poor prognosis [4, 29–34]. In GREEN, patients with an ALC ≥50 × 10^-9^/l, disease bulk ≥5 cm, Binet stage B+C or unmutated IGHV achieved high MRD^–^ remission levels, while lower levels were seen in patients harboring 17p or 11q deletions. The low MRD^–^ and response rates, and shorter PFS in patients with a 17p deletion, reflects the known poor prognosis of this subgroup and emphasizes that these patients need alternative treatment. As numbers in the GREEN analysis were relatively small, the promising MRD^–^ data warrant further investigation of the G-B regimen.

In summary, G-B may represent a new option for previously untreated CLL patients regardless of fitness. Although the non-comparative design of GREEN prevents formal statistical analysis, frontline G-B appeared to have manageable toxicity in fit or unfit patients with CLL. Nonetheless, the TLS rate and associated fatalities highlights the need for careful risk assessment, prophylaxis and monitoring. The low rate of progression and excellent rates of MRD^–^ with G-B indicate that this combination is clinically active. Further follow-up is required to confirm these observations.

## Electronic supplementary material

Stilgenbauer_Green_supplement_revised_clean
