# Supplementary material for: Obinutuzumab plus bendamustine in previously untreated patients with CLL: a subgroup analysis of the GREEN study
Source: Leukemia. 2018 Apr 27;32(8):1778–86. doi: 10.1038/s41375-018-0146-5 (PMC6087718; doi:10.1038/s41375-018-0146-5)
Supplement: Supplementary file 1 — Stilgenbauer_Green_supplement_revised_clean [file 41375_2018_146_MOESM1_ESM.docx]

**Supplementary materials**

**Supplementary methods**

**Patient population**

**Inclusion criteria for cohorts 1, 2, and 3 (previously untreated patients)**

Disease-related inclusion criteria:

1. Previously untreated patients with documented chronic lymphocytic leukemia (CLL) and requiring treatment according to National Cancer Institute (NCI)/ International Workshop on CLL (iwCLL) criteria^1^

2. Patients with 17p deletion and/or p53 mutation could be included at the investigator's discretion

General inclusion criteria:

3. Signed informed consent

4. Age ≥18 years

5. Eastern Cooperative Oncology Group performance status 0–2

6. Life expectancy >6 months according to the investigator’s opinion

7. Adequate hematologic function, defined as follows (unless cytopenia is caused by the underlying disease, i.e. no evidence of additional bone marrow dysfunction [e.g. myelodysplastic syndrome, hypoplastic bone marrow]):

- Hemoglobin ≥9.0 g/dl
- Absolute neutrophil count ≥1.5 × 10^9^/l
- Platelets ≥75 × 10^9^/l
- For patients who will receive bendamustine: leukocyte count >3000/μl

8. Able to comply with study protocol procedures.

**Exclusion criteria**

Disease-related exclusion criteria:

1. Patients who had received more than three previous CLL treatment lines

2. Documented transformation of CLL to aggressive lymphoma (Richter’s transformation)

3. Patients who were refractory to immunochemotherapy

Biochemical and organ function exclusion criteria:

4. Any of the following abnormal laboratory values (unless any of these abnormalities are due to underlying lymphoma):

- Calculated creatinine clearance (CrCl) <30 ml/min (using the Cockcroft–Gault formula)
- Aspartate transaminase or alanine transaminase >2.5 × upper limit of normal (ULN)
- Total bilirubin ≥3 × ULN

5. One or more individual organ/system impairment score of 4 as assessed by the Cumulative Illness Rating Scale (CIRS) definition, excluding the eyes, ears, nose, throat and larynx organ system

General exclusion criteria:

6. Patients with a history of confirmed progressive multifocal leukoencephalopathy

7. History of severe allergic or anaphylactic reactions to monoclonal antibody therapy

8. Known hypersensitivity to the study drugs

9. History of prior malignancy unless the malignancy had been treated with a curative intent and in remission without treatment for ≥5 years prior to enrollment, and with the exception of curatively treated basal cell carcinoma, squamous cell carcinoma of the skin, low-grade in situ carcinoma of the cervix, or low-grade, early-stage localized prostate cancer treated surgically with curative intent

10. Regular treatment (i.e. >5 consecutive days) with corticosteroids during the 28 days prior to the start of cycle 1, day 1, unless administered for indications other than CLL at a dose equivalent to ≤30 mg/d prednisone

11. Regular treatment with immunosuppressive medications following previous organ transplantation

12. Evidence of significant, uncontrolled concomitant diseases that could affect compliance with the protocol or interpretation of results, including significant cardiovascular disease (such as New York Heart Association Class III or IV cardiac disease, severe arrhythmia, myocardial infarction within the previous 6 months, unstable arrhythmias or unstable angina) or pulmonary disease (including obstructive pulmonary disease and history of bronchospasm)

13. Known active bacterial, viral, fungal, mycobacterial, parasitic or other infection (excluding fungal infections of nail beds) or any major episode of infection requiring treatment with intravenous antibiotics or hospitalization (relating to the completion of the course of antibiotics, except if for tumor fever) within 28 days prior to the start of cycle 1, day 1

14. Vaccination with live vaccines within 28 days prior to start of cycle 1, day 1

15. Major surgery (within 28 days prior to the start of cycle 1, day 1), other than for diagnosis

16. Positive test results for chronic hepatitis B infection (defined as positive hepatitis B virus surface antigen [HBsAg] serology) and/or hepatitis B core antibody (HBcAb); patients who have protective titers of hepatitis B surface antibody (HBsAb) after vaccination were eligible

17. Positive test results for hepatitis C (hepatitis C virus [HCV] antibody serology testing)

- Patients positive for HCV antibody were eligible only if polymerase chain reaction was negative for HCV RNA

18. Known history of HIV with seropositive status

19. Positive test results for human T-lymphotropic virus 1 (HTLV-1)

- HTLV testing was required in patients from endemic countries (Japan, countries in the Caribbean basin, South America, Central America, sub-Saharan Africa and Melanesia)

20. Women who were pregnant or lactating

21. Fertile men or women of childbearing potential unless: (1) surgically sterile or (for women) ≥2 years after the onset of menopause; (2) willing to use a highly effective contraceptive method (Pearl Index <1) – such as oral contraceptives, an intrauterine device, sexual abstinence or a barrier method of contraception in conjunction with spermicidal jelly – during study treatment and in female patients for 12 months after the end of antibody treatment and in male patients for 6 months after the end of chemotherapy treatment

22. Participation in another clinical trial with drug intervention within 28 days prior to start of cycle 1, day 1 and during the study.

**Efficacy assessments**

***Treatment response and disease progression.*** ORR was assessed by the investigator per iwCLL criteria^1^ at the ‘final response assessment’ visit (scheduled 84 days after last treatment dose). A computed tomography (CT) scan was required to confirm CR and partial response (PR); responding patients lacking a valid CT scan were classed by the investigator as stable disease (SD). A bone marrow biopsy was required for confirmation of CR; patients otherwise meeting CR criteria but lacking a valid biopsy were classed by the investigator as PR.

PFS was defined as the time from treatment initiation until the first documented progression of disease or death from any cause, whichever occurred first.

***Minimal residual disease.*** MRD measurement was scheduled 84 days after the last dose of study treatment. MRD ‘negativity’ was defined as <1 × 10^-4^ malignant B cells in blood (taken from all patients per protocol) or bone marrow aspirate (taken from patients undergoing bone marrow biopsy to confirm CR), measured by four-color flow cytometry using the international standardized approach,^2^ in a central EuroFlow-certified laboratory based in Kiel, Germany.^3^ Sites unable to ship fresh samples to Kiel within 48 h were prospectively excluded from MRD assessment (remaining sites formed an ‘intent-to-ship’ population [*N* = 140]).

***Prognostic markers*.** Analysis of genomic aberrations by interphase fluorescence in situ hybridization (FISH), evaluation of immunoglobulin heavy variable chain (*IGHV*) mutational status by DNA sequencing, and flow cytometric assessment of CD38 and ZAP70 expression were performed in the central reference laboratories of the German CLL Study Group in Ulm, Cologne and Kiel, respectively.

**Tumor lysis syndrome risk definition and risk minimization measures**

Tumor lysis syndrome (TLS) is an identified risk in patients treated with obinutuzumab and, therefore, guidance and support was provided to investigators before and during the study in relation to the prophylaxis and treatment of TLS. Patients were considered at risk of TLS if they had a peripheral blood lymphocyte count of ≥25 × 10^9^/l or bulky lymphadenopathy. After two reports of fatal TLS cases in patients treated with obinutuzumab–bendamustine (G-B), additional actions were sequentially implemented. Following the first TLS fatality (at that time, the total number of patients enrolled for all cohorts was 513: 326 patients received G-B, of whom 23 reported TLS), additional training of investigators was provided to enhance awareness of TLS risk and management. Following the occurrence of a second fatal TLS event in another cohort of G-B–treated patients from this study (the total number of patients enrolled for all cohorts at that time was 872: 485 patients received G-B, of whom 40 reported TLS), additional risk minimization measures were implemented for G-B–treated patients considered at risk of TLS. These included training of investigators, and a protocol amendment to expand the definition of TLS risk and emphasize the importance of monitoring patients at risk for TLS during cycle 1, as well as additional risk minimization measures, as defined below. These measures were not implemented for patients in cohort 1 (reported here) as they had already completed treatment.

**Expanded risk definition**

An expanded risk definition was used for identifying patients at risk of TLS at baseline prior to treatment with G-B. This definition includes one or more of:

1. Any measurable lymph node ≥10 cm
2. Any measurable lymph node ≥5 cm and <10 cm AND
   - absolute lymphocyte count (ALC) ≥25 × 10^9^/l OR
   - renal impairment defined as CrCl <70 ml/min
3. ALC ≥25 × 10^9^/l AND CrCl <70 ml/min.

**Risk minimization measures**

The following risk minimization measures were highlighted for G-B–treated patients at risk of TLS:

- Oral hydration of 3 liters per day starting 3 days prior to first dose of obinutuzumab, intravenous hydration on day 1 and day 2 of cycle 1, then oral hydration from day 3 to day 8
- Treatment with uricostatics or urate oxidase as per label or local guidance
- Laboratory assessments for TLS prior to infusion of G-B (on days 1, 2 and 8 of cycle 1), and on day 3 and 5 of cycle 1
- Treatment withheld if the Howard criteria for TLS^4^ are fulfilled (two or more electrolyte laboratory abnormalities present simultaneously), or if there is a medically relevant laboratory abnormality in TLS-related parameters or a sign of TLS (e.g. increased serum creatinine or cardiac dysrhythmia), and patients hospitalized and adequately treated until normalization of laboratory abnormalities before treatment is restarted
- Patient education on signs and symptoms of TLS to support reporting
- Additional nephrology consult considered for patients not considered at risk of TLS, but with impaired renal function.

**Sample size**

There was no hypothesis testing and therefore no power calculation as part of this study. The planned sample size of about 950 patients overall allowed the detection of non-frequent AEs (irrespective of grade) e.g. an actual incidence of AEs of 1% could be estimated with a precision of 0.75% (95% CI 0.5–1.9), to be able to obtain an insight into the overall safety profile of obinutuzumab alone and/or in combination with chemotherapy.

**Supplemental tables and figures**

**Table S1. AEs not considered in the manuscript that occurred prior to cut-off but were reported after the data analysis snapshot.**

| AE | Age, years (gender) | Fit/unfit | Onset  (study day) | End (study day) | Grade | SAE | Relationship to obinutuzumab/ chemotherapy | Outcome |
| --- | --- | --- | --- | --- | --- | --- | --- | --- |
| Lesion right ear | 77 (M) | Unfit | NA | 995 | 1 | N | Not related | Resolved |
| Platelets count decreased | 81 (F) | Unfit | 14 | 17 | 2 | N | Related to chemotherapy | Resolved |
| Neutropenia | 67 (F) | Unfit | 166 | NA | 4 | N | Not related | Lost to follow-up |
| Multiple injuries* | 72 (M) | Unfit | 975 | 1215 | 3 | Y | Not related | Resolved with sequelae |
| Fall* | 72 (M) | Unfit | 975 | 975 | 3 | N | Not related | Resolved with sequelae |

Abbreviations: AE, adverse event; F, female; M, male; N, no; NA, not available; SAE, serious adverse event; Y, yes.

*AEs occurred in the same patient.

**Table S2. Change in final response assessment.***

|  | Age, years  (gender) | Fit/unfit | Primary analysis snapshot | Updated to |
| --- | --- | --- | --- | --- |
| **Response** | 64 (M) | Unfit | SD | PR |

Abbreviations: M, male; PR, partial response, SD, stable disease.

*After the data analysis snapshot was taken, one response was changed from SD to PR by a site on the database that remained open to continue collecting information until the final analysis.

**Table S3. Further details of fatal adverse events in patients receiving G-B in cohort 1 of GREEN by subgroup**

| Patient | Age, years (gender) | Fit/ unfit | Days since last treatment dose for G/B | Reason for death by AE preferred term | Study day of death* | PD before AE (study day) | AE details | |
| --- | --- | --- | --- | --- | --- | --- | --- | --- |
|  |  |  |  |  |  |  | Related to study drug G/B^†^ | Study day of AE onset |
| 1 | 67 (F) | Unfit | 49/48 | Brain abscess | 168 | N | N/N | 110 |
| 2 | 77 (M) | Unfit | 260/259 | Pneumonia | 401 | N | N/N | 397 |
| 3 | 79 (M) | Unfit | 12/12 | TLS and febrile neutropenia | 13 | N | Y/Y^‡^ | 7 (TLS)  12 (febrile neutropenia) |
| 4 | 59 (F) | Fit | 53/52 | West Nile viral infection | 145 | N | Y/Y | 109 |
| 5 | 71 (M) | Fit | 31/31 | Acute hepatic failure | 59 | N | Y/Y | 55 |
| 6 | 55 (F) | Unfit | 57/56 | Lung adenocarcinoma metastatic | 113 | N | N/N | 78 |
| 7 | 75 (M) | Unfit | 649/648 | Pneumothorax | 792 | N | N/N | 785 |
| 8 | 71 (M) | Fit | 236/235 | Gastric adenocarcinoma | 376 | N | N/N | 240 |
| 9 | 77 (M) | Unfit | 7/20 | Arrhythmia | 21 | N | Y/Y | 21 |
| 10 | 65 (F) | Fit | 573/572 | Pneumonia | 721 | Y (267) | N/N | 674 |
| 11 | 49 (F) | Unfit | 327/326 | Sepsis | 459 | N | N/N | 459 |
| 12 | 74 (M) | Unfit | 329/328 | Adenocarcinoma | 420 | Y (186) | Y/Y | 285 |
| 13 | 70 (M) | Fit | 576/575 | Squamous cell carcinoma of skin | 716 | N | N/N | 572 |

Abbreviations: AE, adverse event; F, female; G-B, obinutuzumab–bendamustine; M, male; N, no; PD, progressive disease; TLS, tumor lysis syndrome; Y, yes.

*Study day is relative to the day of first administration of any study treatment. Day of first administration of any study treatment is defined as day 1.

^†^Causality as per investigator.

^‡^For both AEs**.**

**Table S4. Progression-free survival in patients receiving G-B in cohort 1 of GREEN by subgroup (intent-to-treat population)**

| Subgroup | Status | Patients per group | Number of events | 1-year PFS, %* | 2-year PFS, %* |
| --- | --- | --- | --- | --- | --- |
|  |  |  |  |  |  |
| All patients |  | 158 | 37 | 92.31 | 81.77 |
| Fitness status | Fit | 70 | 17 | 94.20 | 82.51 |
|  | Unfit | 88 | 20 | 90.80 | 81.19 |
| MRD status in blood | Negative | 94 | 15 | 100.00 | 90.25 |
|  | Positive | 9 | 5 | 88.89 | 55.56 |
| Age | <65 years | 49 | 10 | 95.74 | 87.13 |
|  | ≥65 years | 109 | 27 | 90.83 | 79.44 |
| Gender | Male | 103 | 25 | 93.14 | 83.10 |
|  | Female | 55 | 12 | 90.74 | 79.26 |
| IGHV | Mutated | 44 | 6 | 90.70 | 85.85 |
|  | Unmutated | 92 | 28 | 93.41 | 78.92 |
| CD38 | Positive | 70 | 17 | 92.86 | 78.44 |
|  | Negative | 59 | 13 | 96.49 | 87.41 |
| ZAP70 | Positive | 82 | 20 | 96.34 | 83.95 |
|  | Negative | 47 | 10 | 91.11 | 79.75 |
| Genomic aberrations | 17p deletion | 11 | 5 | 63.64 | 54.55 |
|  | 11q deletion | 26 | 11 | 96.00 | 67.20 |
|  | 12q trisomy | 26 | 9 | 92.31 | 72.87 |
|  | 13q deletion | 52 | 6 | 94.12 | 89.93 |
|  | Other | 6 | 0 | 100.00 | 100.00 |
|  | No abnormality | 25 | 4 | 96.00 | 96.00 |

Abbreviations: G-B, obinutuzumab–bendamustine; IGHV, immunoglobulin heavy variable chain; MRD, minimal residual disease; PFS, progression-free survival.

*Kaplan–Meier estimates.

**Figure S1. Study design.**


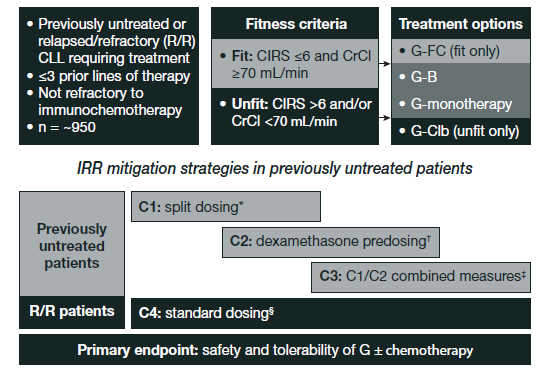


- Pre-medication with a steroid (prednisolone or equivalent, not hydrocortisone; 1 h pre-dosing), antihistamine (~30 min pre-dosing) and acetaminophen (~30 min pre-dosing) was mandated before the first administration of G (C1 D1 and D2).
- Chemotherapy options were fludarabine and cyclophosphamide for fit patients (CIRS ≤6 and CrCl ≥70 ml/min), chlorambucil for unfit patients (CIRS >6 and/or CrCl <70 ml/min), bendamustine for fit/unfit patients, or obinutuzumab monotherapy.
- For previously untreated patients in the cohort 1 subgroup, bendamustine was administered i.v. at a dose of 90 mg/m^2^ over a 60-min period on D1 and D2 of C1–C6. A reduced dose of bendamustine 70 mg/m^2^ was available for unfit patients at the investigators’ discretion.
- The primary endpoint was safety and tolerability. Secondary endpoints included overall response rate (investigator assessed) including complete response, minimal residual disease and progression-free survival.

**25 mg G on C1 D1 at 12.5 mg/h and 975 mg G on C1 D2 at 50 mg/h* then 1000 mg G on D8 and D15 of C1, and D1 of C2–6 as standard. ^†^100 mg G on C1 D1 at 25 mg/h and 900 mg G on C1 D2 at 50 mg/h *with oral dexamethasone 20 mg or equivalent 12 h pre-dose,* then 1000 mg G on D8 and D15 of C1, and D1 of C2–6 as standard. ^‡^*25 mg G on C1 D1 at 12.5 mg/h and 975 mg G on C1 D2 at 50 mg/h* *with oral dexamethasone 20 mg or equivalent 12 h pre-dose,* then 1000 mg G on D8 and D15 of C1, and D1 of C2–6 as standard*.* ^§^100 mg G on C1 D1 at 25 mg/h and 900 mg G on C1 D2 at 50 mg/h, then 1000 mg G on D8 and D15 of C1, and D1 of C2–6 at 100 mg/h (escalated in increments of 50 mg/h every 30 min to a maximum rate of 400 mg/h in the absence of infusion-related reactions/hypersensitivity). C, cycle; CIRS, Cumulative Illness Rating Scale; CLL, chronic lymphocytic leukemia; CrCl, creatinine clearance; D, day; G, obinutuzumab monotherapy; G-B, obinutuzumab–bendamustine; G-Clb, obinutuzumab–chlorambucil; G-FC, obinutuzumab–fludarabine–cyclophosphamide; IRR, infusion-related reaction; i.v., intravenously; R/R, relapsed/refractory.

**Figure S2. Patient disposition.**


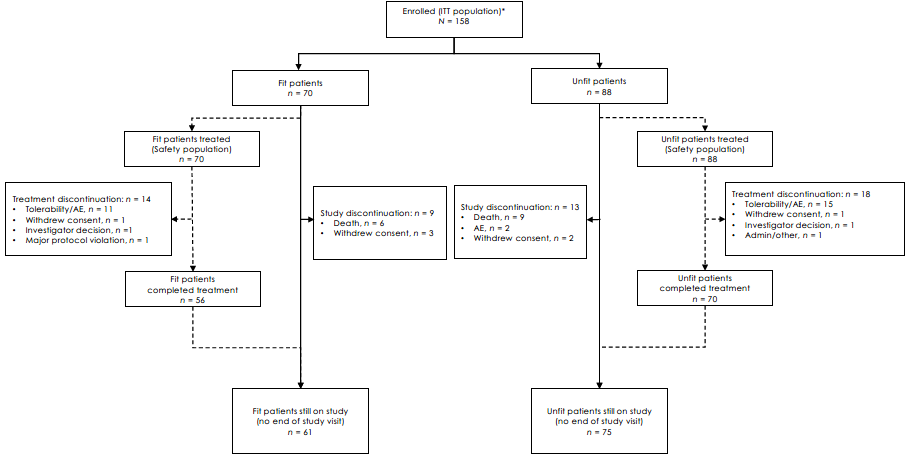


*Intent-to-treat: all patients from cohort 1 who received at least a partial dose of both obinutuzumab and bendamustine. AE, adverse event.

**References**

1. Hallek M, Cheson BD, Catovsky D, Caligaris-Cappio F, Dighiero G, Döhner H *et al*. Guidelines for the diagnosis and treatment of chronic lymphocytic leukemia: a report from the International Workshop on Chronic Lymphocytic Leukemia updating the National Cancer Institute-Working Group 1996 guidelines. *Blood* 2008; **111**; 5446–5456.
2. Rawstron AC, Villamor N, Ritgen M, Böttcher S, Ghia P, Zehnder JL *et al*. International standardized approach for flow cytometric residual disease monitoring in chronic lymphocytic leukaemia. *Leukemia* 2007; **21**: 956–964.

Kalina T, Flores-Montero J, van der Velden VH, Martin-Ayuso M, Böttcher S, Ritgen M *et al*; EuroFlow Consortium (EU-FP6, LSHB-CT-2006-018708). EuroFlow standardization of flow cytometer instrument settings and immunophenotyping protocols. *Leukemia* 2012; **26**: 1986–2010.

1. Howard SC, Jones DP, Pui CH. The tumour lysis syndrome. *N Engl J Med* 2011; **364**: 1844–1854.
